# Epigenetic landscapes of intracranial aneurysm risk haplotypes implicate enhancer function of endothelial cells and fibroblasts in dysregulated gene expression

**DOI:** 10.1186/s12920-021-01007-9

**Published:** 2021-06-16

**Authors:** Kerry E. Poppenberg, Haley R. Zebraski, Naval Avasthi, Muhammad Waqas, Adnan H. Siddiqui, James N. Jarvis, Vincent M. Tutino

**Affiliations:** 1 Canon Stroke and Vascular Research Center, University at Buffalo, Clinical and Translational Research Center, 875 Ellicott Street, Buffalo, NY 14214 USA; 2 Department of Neurosurgery, University at Buffalo, Buffalo, NY USA; 3 Department of Biomedical Engineering, University at Buffalo, Buffalo, NY USA; 4 Department of Pediatrics, University at Buffalo, Buffalo, NY USA; 5 Department of Pathology and Anatomical Sciences, University at Buffalo, Buffalo, NY USA; 6 Department of Mechanical and Aerospace Engineering, University at Buffalo, Buffalo, NY USA

**Keywords:** Intracranial aneurysm, Epigenetics, Genetic risk, Histone mark, Topologically associated domain

## Abstract

**Background:**

Genome-wide association studies have identified many single nucleotide polymorphisms (SNPs) associated with increased risk for intracranial aneurysm (IA). However, how such variants affect gene expression within IA is poorly understood. We used publicly-available ChIP-Seq data to study chromatin landscapes surrounding risk loci to determine whether IA-associated SNPs affect functional elements that regulate gene expression in cell types comprising IA tissue.

**Methods:**

We mapped 16 significant IA-associated SNPs to linkage disequilibrium (LD) blocks within human genome. Using ChIP-Seq data, we examined these regions for presence of H3K4me1, H3K27ac, and H3K9ac histone marks (typically associated with latent/active enhancers). This analysis was conducted in several cell types that are present in IA tissue (endothelial cells, smooth muscle cells, fibroblasts, macrophages, monocytes, neutrophils, T cells, B cells, NK cells). In cell types with significant histone enrichment, we used HiC data to investigate topologically associated domains (TADs) encompassing the LD blocks to identify genes that may be affected by IA-associated variants. Bioinformatics were performed to determine the biological significance of these genes. Genes within HiC-defined TADs were also compared to differentially expressed genes from RNA-seq/microarray studies of IA tissues.

**Results:**

We found that endothelial cells and fibroblasts, rather than smooth muscle or immune cells, have significant enrichment for enhancer marks on IA risk haplotypes (*p* < 0.05). Bioinformatics demonstrated that genes within TADs subsuming these regions are associated with structural extracellular matrix components and enzymatic activity. The majority of histone marked TADs (83% fibroblasts [IMR90], 77% HUVEC) encompassed at least one differentially expressed gene from IA tissue studies.

**Conclusions:**

These findings provide evidence that genetic variants associated with IA risk act on endothelial cells and fibroblasts. There is strong circumstantial evidence that this may be mediated through altered enhancer function, as genes in TADs encompassing enhancer marks have also been shown to be differentially expressed in IA tissue. These genes are largely related to organization and regulation of the extracellular matrix. This study builds upon our previous (Poppenberg et al., BMC Med Genomics, 2019) by including a more diverse set of data from additional cell types and by identifying potential affected genes (i.e. those in TADs).

**Supplementary Information:**

The online version contains supplementary material available at 10.1186/s12920-021-01007-9.

## Background

Intracranial aneurysms (IAs) are focal dilations of the cerebral vessels that are present in approximately 2–6% of the general population [1]. IA is a complex disease trait, in which heritable genetic variants are known to play an important role [2]. Indeed, family history of IA has been shown to be a significant risk factor for developing an aneurysm (odds ratio-OR 4) [3]. In studying genetic risk for IA, genome-wide association studies (GWAS) have identified many aneurysm-associated single nucleotide polymorphisms (SNPs) [4–12], the most significant existing at 2q32.1 (*PLCL1*) [12], 8q11.23–q12.1 (*SOX17*) [12], 9p21.3 (*CDKN2A-CDKN2B*) [12], 18q11.2 (*RBBP8*) [10], 13q13.1 (*STARD13*) [10], and 10q24.32.12 [10]. Notably, many IA-risk loci include functional, noncoding regions, suggesting that genetic risk may operate on these regulatory elements, including enhancers [13], that influence gene expression, rather than on the structure of the gene product itself [14]. However, deriving mechanistic insights from GWAS data alone is problematic, since the true variant(s) that confer risk are not usually known. SNPs used to tag risk loci on GWAS are typically in linkage disequilibrium (LD) with dozens or hundreds (sometimes even thousands) of other SNPs, most of which have no influence on disease risk [15–17]. One way to gain insight into mechanisms through which genetic variants associated with IA exert risk is to examine the chromatin structures across the entire haplotypes.

In a recent study by our group [18], we explored the chromatin landscapes associated with the haplotypes encompassing 16 SNPs strongly associated with IA that were reported in a meta-analysis by Alg et al. [19]. Using ChIP-seq data available from the ENCODE project, we queried the LD blocks that incorporated the relevant tag SNPs in endothelial cells (ECs), monocytes, neutrophils, and peripheral blood mononuclear cells (PBMCs) to determine whether there was epigenetic evidence that genetic risk was conferred more predominantly in circulating immune cells or the vascular endothelium [18]. From this data, we found that the cells of the endothelium, rather than immune cell types, were statistically significantly enriched (compared to genome background) for H3K4me1and H3K27ac histone marks, epigenetic features usually associated with functional enhancers. Enhancers, noncoding regulatory elements that play an important role in modulating gene expression, serve as regulators that fine-tune gene expression to fit specific physiologic contexts [20]. Thus, our data suggest that known genetic risk factors for IA may alter gene expression in the vessel wall through modulation of enhancer activity. Yet, it is known that the presence of H3K4me1 and H3K27ac at specific genomic locations is not sufficient to establish that the region in question truly has enhancer activity and is thus involved in active gene transcription. The locations of the marks are also not sufficient to determine the specific genes that might be affected, as it is widely known that enhancers can regulate more than one gene, and may not regulate the gene(s) in closest proximity.

In this study, we sought to gain further insight into how IA risk loci impinge on noncoding, regulatory elements and influence gene expression by interrogating additional chromatin features that encompass the established IA risk loci. To do this, we took advantage of the fact that chromatin is organized in distinct DNA looping structures, or topologically associated domains (TADs), that are demarcated by the presence of the anchoring structures CTCF and cohesion. As Gasperini et al. detailed, enhancers do not always influence the nearest gene in terms of linear genomic distance, but almost invariably regulate genes within the same TAD [21]. Seeing as our previous analysis demonstrated that the vascular tissue may confer genetic risk for IA, we examined histone marks and their associated TADs that subsume IA-associated LD blocks in cells present in IA tissue (specifically ECs [22], B cells [23], fibroblasts [22], macrophages and monocytes [22, 23], natural killer (NK) cells [24], neutrophils [22, 25], smooth muscle cells (SMCs) [22, 26], and T cells [22, 23]). After assessing histone marks in each cell type, we investigated likely enhancer targets by querying the overlap with TAD data derived from HiC experiments [27] and performing bioinformatics analyses on genes found within the TADs. If genetic variants are operating on genes in these TADs, we might expect them to be differentially expressed in the IA tissue. To test this idea, we compared identified target genes within HiC-defined TADs with differentially expressed genes reported from RNA sequencing or gene chip microarray studies performed on human IA tissue.

## Methods

### Identification of LD blocks

We analyzed 16 of 19 IA-associated SNPs identified in a meta-analysis by Alg et al. [19] to have been reported as significantly associated with IA in two or more previous publications. We note that SNPs identified on GWAS do not identify the causal polymorphism, rather, they index a larger genetic region where risk may operate. Therefore, the causal SNP may be anywhere within the LD block. For this reason, we examined the haplotype blocks (or LD blocks) surrounding the index SNPs of interest, as these regions are inherited together, with an r-squared of 0.9. We used the UCSC LiftOver tool (https://genome.ucsc.edu/cgi-bin/hgLiftOver) to identify the LD block positions in hg38 for use in our histone enrichment analysis.

### Identification of H3K4me1/H3K27ac/H3K9ac histone marks within LD blocks

We used the Cistrome data browser to find hg38 ChIP-Seq peak data for H3K27ac, H3K4me1, and H3K9ac histone marks [28, 29]. All data from Cistrome is processed using ChiLin pipeline [30], which uses BWA to align raw data and MACS2 to call peaks. Cistrome also reports 7 quality control metrics for each dataset, including sequence quality and ChIP enrichment. We specifically investigated cell types found in the aneurysm walls, as reported by histological studies of human IA tissues [23, 31–33]. These included human B lymphocytes, ECs (aortic, HAEC, and umbilical vein, HUVEC), fibroblasts (skin and lung), macrophages, monocytes, NK cells, neutrophils, SMCs, and T lymphocytes (CD4+ and CD8+). For each, we selected peaks files from the ENCODE consortium or those with high quality scores. Additional file 1: Table S1 reports the data source and GEO or ENCODE accession number for each of the data files used. Accession numbers also listed in availability of data and materials section. To identify if any of the histone peaks overlapped with our LD blocks of interest, we used the intersect command within the BEDTools command line software.

To test for significance enrichment of the marks, we empirically calculated the *p* value using the z-score from a histogram based on 1000 iterations. In brief, we first created 16 random regions from hg38 genome file with an average length equal to the average length of the IA-associated LD blocks. We then used the intersect command to determine how many of the 16 random regions overlapped with the peaks for the various cell types’ histone marks. A histogram was created to depict the distribution over all 1000 iterations and to calculate the associated mean (μ) and standard deviation (σ). We calculated the z-score using the following equation: $z=\frac{x-\mu }{\sigma }$ in which x is the number of intersections between the IA-associated LD blocks and the peak file of interest. The *p* value was then determined from the z-score; *p* value < 0.05 was deemed significant. For cell types with significant H3K27ac marks, we also queried H3K9me3 marks, which indicate that chromatin is closed and inaccessible.

### Identification of TADs

To identify TADs encompassing the IA-associated risk haplotypes, as well as the genes within these loop structures, we mined existing HiC data available on the 3D Genome browser [34]. HiC is a non-directed technique that allows one to discern pairwise contacts between any two regions in the genome based on their physical proximity [35]. For cell types in which there were significant association with enhancer marks, all genes encompassed within the TADs subsuming the regions with significant enhancer marks were recorded and used in bioinformatics analyses.

### Bioinformatics

For genes identified within the TADs that encompass the IA risk haplotypes in which there were significant association with H3K9ac/H3K4me1/H3K27ac marks, we performed gene ontology enrichment (GO) analysis via g:Profiler under the default settings. GO terms were reported if the input gene list was enriched for any term to a greater degree than what would be expected by chance (adjusted *p* value < 0.05) [36]. The genes within the relevant TADs were also analyzed using Ingenuity Pathway Analysis (IPA) [37]. Here, each gene identifier was mapped to its corresponding gene object in the Ingenuity Knowledge Base and overlaid onto a molecular network derived from information accumulated in the Knowledge Base. Related disease or function annotations with a Benjamini–Hochberg *p* value < 0.01 that were identified using at least 3 of the input genes were deemed significant. Gene networks were algorithmically generated based on their “connectivity” derived from known interactions between the products of the input genes. Networks were considered significant if their p-scores were > 21.

### Comparison with IA tissue differential expression studies

In order to gather further evidence that enhancers on the risk haplotypes influence gene expression in the cell types of interest, we compared the genes from IA-associated TADs with differentially expressed genes from IA tissue studies. The rationale for this approach is the strong experimental evidence that enhancers almost invariably regulate genes within the same TAD [21]. Thus, we sought to determine whether there were any likely candidate genes whose expression levels might be impacted by genetic variants within the H3K4me1/H3K27ac/H3K9ac-marked regions. We included 8 studies that investigated differential expression between aneurysm and control tissues or between ruptured IAs and unruptured IAs. Differentially expressed genes between IA and control tissues were reported by Aoki [38], Kleinloog [39], Li [40], Pera [41], Shi [42], and Wang [43]. Differential expression between ruptured and unruptured IAs was investigated by Kleinloog [39], Kurki [44], Nakaoka [45], and Pera [41]. We compared their published lists of differentially expressed genes (DEGs) with those found in IA-associated TADs.

## Results

### Queried LD blocks

In this study, we analyzed the epigenetic landscapes of 16 LD blocks, which are reported in Table 1 (Additional file 1: Table S2 also reports both hg19 and hg38 positions for the LD blocks, as they were originally identified in hg19 but mapped to hg38 to agree with Cistrome data). Based on our analysis, we found that the majority (12/16) of the sentinel SNPs were located within noncoding regions, as is common in complex traits. The remaining 4 index SNPs were found to be within exons, although it cannot be assumed that the SNP impacts the coding function of the given gene [46].

**Table 1:** IA risk associated SNPs and the associated linkage disequilibrium blocks

Sentinel SNP	SNP Location	Nearest Gene	LD Block (hg38)	Length (bp)
rs3767137	Intronic	*HSPG2*	chr1:21834230–21841817	7587
rs1800255	Exonic	*COL3A1*	chr2:188976887–189003156	26,269
**rs1429412**	Intergenic	*ANKRD44*	chr2:197283467–197358397	74,930
rs700651	Intronic	*BOLL*	chr2:197676674–197766990	90,316
rs6841581	Intergenic	*EDNRA*	chr4:147444187–147493499	49,312
rs251124	Intronic	*VCAN*	chr5:83509605–83530435	20,830
rs4628172	Intronic	*AGMO*	chr7:15454259–15466904	12,645
**rs1800796**	Intronic	*IL6*	chr7:22726627–22732119	5492
rs42524	Exonic	*COL1A2*	chr7:94413927–94420044	6117
*rs10958409*	Intergenic	*–*	chr8:54397171–54415556	18,385
rs9298506	Intergenic	*–*	chr8:54509054–54549764	40,710
rs2891168	Intronic	*CDKN2B-AS1*	chr9:22072265–22125504	53,239
**rs10757278**	Intergenic	*CDKN2B-AS1*	chr9:22077086–22125504	48,418
**rs6538595**	Intronic	*FGD6*	chr12:95095355–95123067	27,712
rs4934	Exonic	*SERPINA3*	chr14:94612340–94614466	2126
rs1132274	Exonic	*RRBP1*	chr20:17613385–17619469	6084

**Fig. 1:**
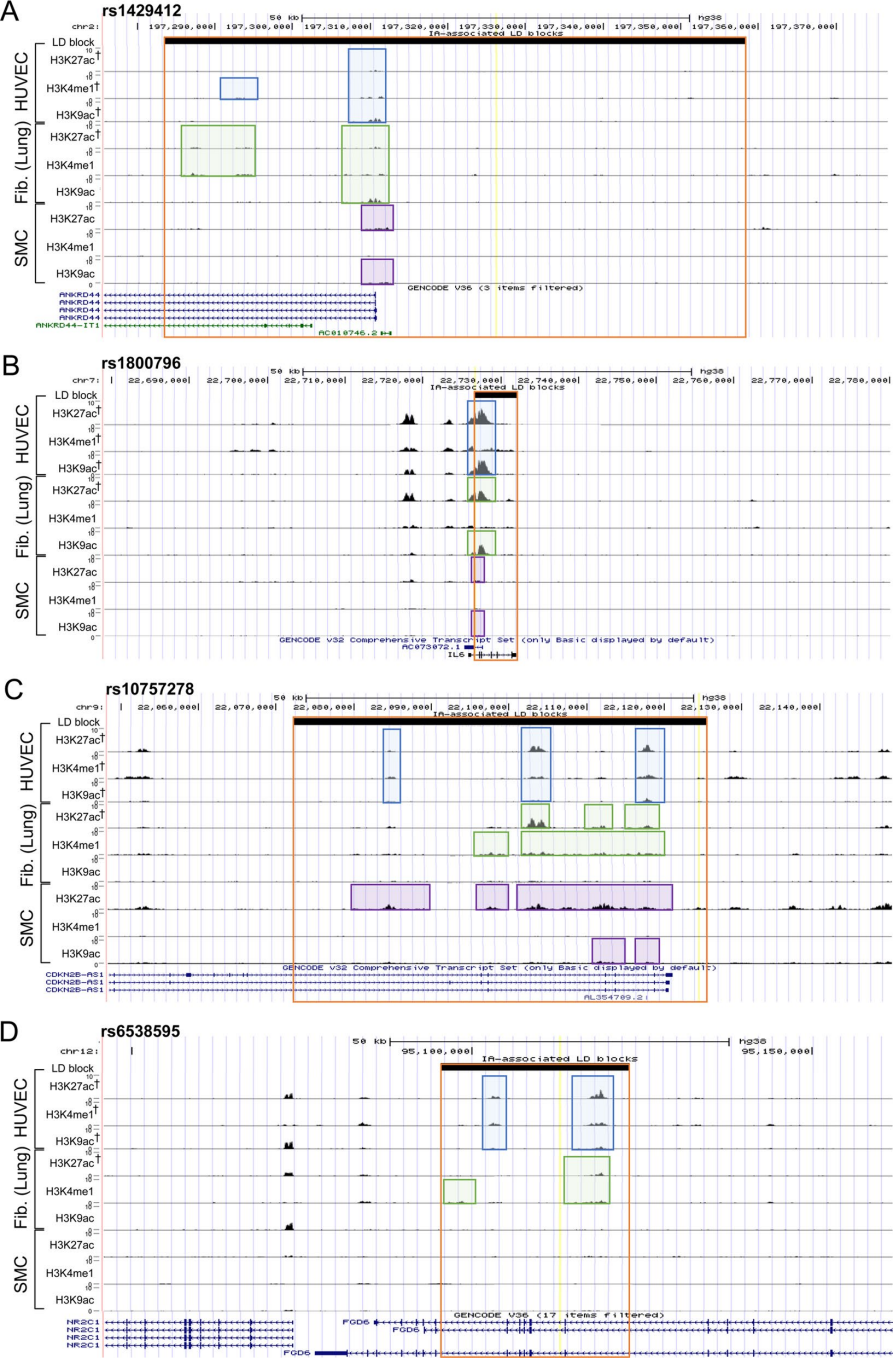
Landscape around four example IA-associated LD blocks showing enhancer marks in three cell types. HUVECs in blue, fibroblasts (lung) in green, SMCs in purple. IA-associated LD block in orange, index SNP is yellow line. “†” denotes significance. Relative height of ChIP-seq peaks all scaled from 0–10 in Genome Browser. **A **Genome browser screenshot showing the IA-associated LD block identified by the intergenic tap SNP rs1429412 near *ANKRD44*. All three marks (H3K27ac, H3K4me1, and H3K9ac) were present for both HUVECs and fibroblasts. **B** Genome browser screenshot showing the IA-associated LD block for the IL6 locus identified by the intronic tag SNP, rs1800796. All three marks are present for HUVECs. **C** Genome browser screenshot showing the IA-associated LD block identified by the intergenic tag SNP rs10757278 near *CDKN2B-AS1*. All three marks were present for HUVECs. **D** Genome browser screenshot showing the IA-associated LD block for the *FGD6* locus identified by intronic tag SNP, rs6538595. All three marks were present for HUVECs

**Fig. 2:**
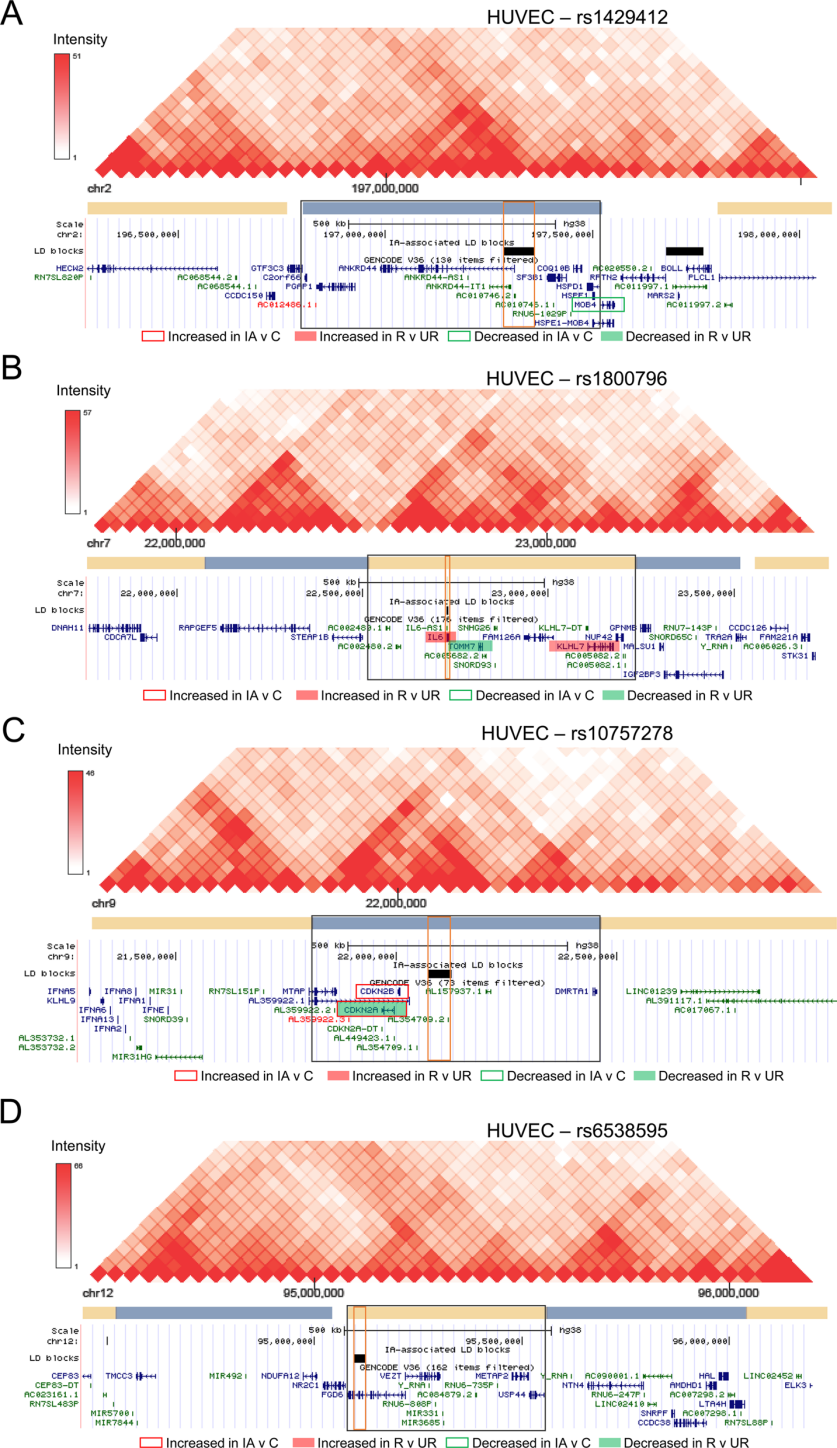
Visualization of the topologically associated domain (TAD) of four example SNPs in HUVECs. Haplotype outlined in red. TAD outlined in black. Genes reported as differentially expressed in IA tissue studies are highlighted in red (increased expression) or green (decreased expression). Border indicates identified in IA vs control; fill indicates identified in ruptured vs unruptured IAs. **A** HiC data surrounding rs1429412 shows a large TAD in HUVECs. **B** Data from HiC analysis shows another large TAD surrounding rs1800796. **C** HiC data shows the TAD surrounding rs10757278. **D** HiC map showing TAD encompassing rs6538595 does not contain any genes found to be differentially expressed in IA tissue studies

Bolded SNPs were chosen as examples in Fig. 1, Fig. 2, and Additional file 1: Fig. S1

hg, human genome; IA, intracranial aneurysm; rs, reference SNP cluster ID; SNP, single nucleotide polymorphism; chr, chromosome; LD, linkage disequilibrium

### Histone marks for enhancers are significantly enriched in endothelial cells and fibroblasts

We investigated epigenetic features associated with enhancer function in the LD blocks for a variety of cell types known to be present in the aneurysmal tissue (B cells, ECs, fibroblasts, macrophages, monocytes, NK cells, neutrophils, SMCs, T cells) using data from the Cistrome browser. HUVECs demonstrated significant enrichment for histone marks in IA-associated LD blocks as compared to the randomly-generated genomic regions (H3K4me1 *p* = 0.00025, H3K9ac *p* = 0.014, H3K27ac *p* = 0.0031), consistent with our previously published results [18]. As shown in Table 2, 7 of the 16 queried LD blocks had enrichment for all 3 histone marks in HUVEC data. While only H3K27ac data was available for aortic ECs, ChIP-seq peaks were present in 8 regions, which was significant (*p* = 0.011). Skin fibroblasts also demonstrated significant enhancer mark enrichment (H3K4me1 *p* = 0.0091, H3K9ac *p* = 0.010, H3K27ac *p* = 0.0027) and had all 3 marks in 5 LD blocks. NK cells and T lymphocytes had the fewest H3K4me1/H3K27ac/H3K9ac peaks of the cell types examined.

**Table 2:**
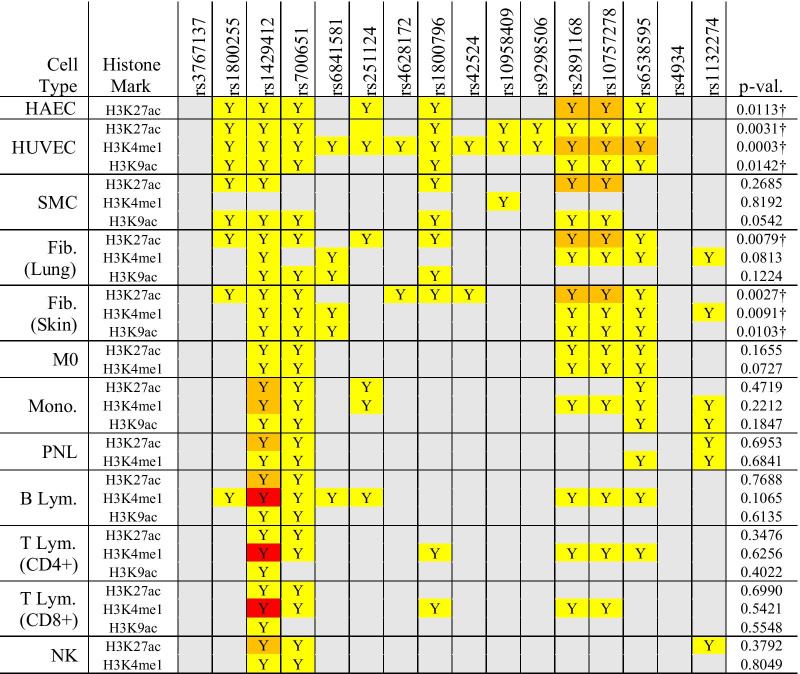
Histone marks present in IA risk associated linkage disequilibrium blocks

The color of each cell indicates number of peaks in each region with enrichment (grey = none, yellow = 1–5, orange = 6–10, red = 11 or more)

“†” denotes significance

Fib., fibroblast; HAEC, human aortic endothelial cell; HUVEC, human umbilical vein endothelial cell; IA, intracranial aneurysm; lym., lymphocyte; M0, macrophage; mono., monocyte; NK, natural killer cell; p-val., p-value; PNL, polymorphonuclear leukocyte; rs, reference SNP cluster ID; SMC, smooth muscle cell; Y, yes

We examined H3K9me3 marks in those cell types with significant marks, namely HUVECs, lung fibroblasts, and skin fibroblasts (accession numbers provided in availability of data and materials section and Additional file 1: Table S1). While HAEC had significant H3K27ac marks, there was no H3K9me3 dataset of sufficient quality to examine. None of the haplotypes queried encompassed H3K9me3 marks.

For example, we show the landscape around a large LD block associated with the intergenic SNP rs1429412, which is nearest to *ANKRD44*, in Fig. 1A. This region was particularly rich containing all marks for all cell types except for H3K4me1 in SMCs. Another example containing H3K4me1/H3K27ac/H3K9ac peaks in ECs and fibroblasts was centered around rs1800796, which falls within the first intron of IL6, an important inflammatory cytokine that has been strongly implicated in IA pathogenesis (Fig. 1B) [47]. H3K27ac marks were prominent in HAECs, HUVECs, fibroblasts (lung and skin), and SMCs. Both HUVECs and T cells (CD4 + and CD8 +) expressed H3K4me1 marks within this haplotype. H3K9ac marks within this LD block were found in HUVECs, fibroblasts (lung), and SMCs. In this LD block, HUVECs were the only cell type to express the 3 queried histone marks. A similar pattern of histone mark enrichment was also present in the epigenetic landscape around rs10757278, an intergenic region flanking *CDKN2B-AS1* (Fig. 1C). H3K27ac marks were prominent in HAECs, HUVECs, fibroblasts (lung and skin), SMCs, and macrophages. HUVECs, fibroblasts, macrophages, monocytes, and lymphocyte populations had H3K4me1 marks present within this haplotype, while the H3K9ac marks were found only in HUVECs, skin fibroblasts, and SMCs. HUVECs and skin fibroblasts both expressed all 3 queried histone marks in this block. In Fig. 1D, we present the landscape surrounding an intronic SNP for *FGD6*, rs6538595. All marks were present in HUVECs, skin fibroblasts, and monocytes, while H3K27ac and H3K4me1 marks were present in lung fibroblasts and macrophages. Neutrophils, B cells, and CD4 + T cells contained H3K4me1 marks. Figure 1 provides visualization of data reported in Table 2 for four example regions (bolded SNPs in Table 1). Landscapes surrounding other SNPs can be visualized in UCSC Genome Browser by loading data from Cistrome.

### Genes in IA-associated TADs reflect ontologies related to extracellular matrix

For the IA-associated LD blocks with histone marks present, we identified TADs and the genes encompassed within them. While TAD boundaries have been found to be conserved [48, 49], there is evidence suggesting TADs do vary among cell types [27, 50, 51]. Therefore, we examined HUVEC and fibroblast TADs independently using HiC data from Rao et al. [27]. Because HiC data were unavailable for skin fibroblasts, we used data from IMR90 cells (fetal lung fibroblasts). Figure 2 demonstrates the TADs that surround our four example SNPs, rs1429412 (A), rs1800796 (B), rs10757278 (C), and rs6538595 (D) in HUVECs. The TADs for these same four regions in fibroblasts (IMR90 cells) are presented in Additional file 1: Fig. S1. We found 164 transcripts within the TADs for HUVECs and 153 transcripts within the IMR90 TADs. See Additional file 1: Table S3 for genes encompassed within each TAD. Between the two cell types, 114 transcripts were common, and many were related to structural components, such as *COL1A2*, *COL3A1*, *HAPLN1*, *VCAN*, and *VEZT*. The g:Profiler showed that in HUVECs, 1 biological process GO term, 3 cellular component GO terms, and 7 molecular function GO terms were significantly enriched (see Additional file 1: Table S4). These annotations demonstrate enrichment of lipoxygenase pathway, fibrillar collagen trimer, and extracellular matrix structural constituent and peptidase regulator function. Fewer ontologies were associated with fibroblast (IMR90) TAD genes, with only 2 significant GO terms (S-methyl-5-thioadensoine phosphorylase activity and purine-nucleoside phosphorylase activity).

**Fig. 3:**
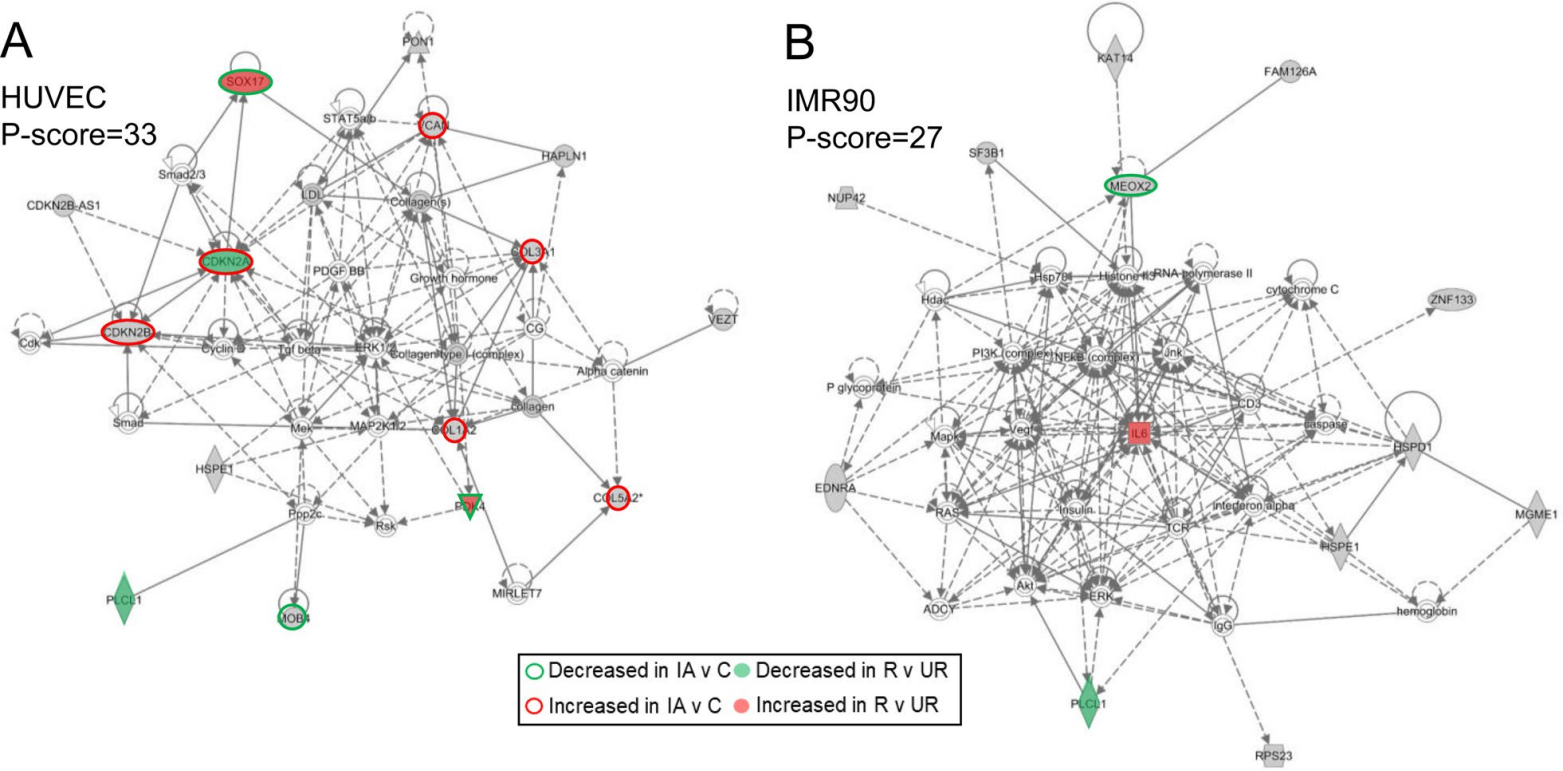
Top IPA networks of transcripts in TADs encompassing predicted enhancers in HUVECs and fibroblasts. Transcripts present in TADs are in grey and lines indicate interactions (solid = direct, dashed = indirect). Genes reported as differentially expressed in IA tissue studies are highlighted in red (increased expression) or green (decreased expression). Border indicates identified in IA vs control; fill indicates identified in ruptured vs unruptured IAs. **A** For HUVECs, the top network with a p-score of 33 showed hubs at CDKN2A/B, ERK1/2, and collagens. **B** In fibroblasts, the top network with a p-score  of 27 demonstrated interactions at IL6 and NF-kB

We used IPA to further characterize disease and biological functions and to investigate potential gene interaction networks based on genes found in the TADs with histone mark enrichment. For HUVECs, there were 13 significantly enriched diseases and bio-functions, including vascular lesion, Loeys-Dietz syndrome, and Ehlers-Danlos syndrome (the full list of significant terms is presented in Additional file 1: Table S5). Network analysis showed 2 significant networks with P-scores of 33 and 30, respectively, which were populated by connections around collagens and *ERK1/2* genes (first network) and connections around ELAVL1 and TP53 (second network). Generally, these networks were related to connective tissue disorders and cellular assembly and organization. For TAD genes associated in fibroblasts (IMR90), IPA did not return any significant disease and biological function annotations. However, there were 3 significant networks with P-scores of 27, 25, and 25, which had connections emanating from hubs of NF-KB and IL6 (first network), from hubs of MAPK and ERK (second network), and from hubs of ESR2 and TGFB1 (third network). The top network associated with each cell type is presented in Fig. 3 (A- HUVECs, B- fibroblasts); the remaining networks are provided in Additional file 1: Fig. S2. Broadly, these networks were related to nucleic acid metabolism, dermatological diseases and conditions, and cellular growth and proliferation. See Additional file 1: Table S6 for additional details on the networks described above.

### Genes in IA-associated TADs overlap with those differentially expressed in IA tissue

We compared the genes within the HUVEC and IMR90 TADs surrounding the IA SNPs of interest to genes differentially expressed in aneurysm tissue, reported in 8 distinct studies (see Additional file 1: Table S7 for information related to these publications). In HUVECs, 11/13 (85%) IA-associated TADs enriched for H3K4me1/H3K27ac/H3K9ac histone marks also contained a differentially expressed gene in IA tissue. Similarly, 10/12 (83%) TADs encompassed a tissue DEG in IMR90 cells. (See Additional file 1: Table S3). We first analyzed the tissue studies that compared IA tissue to control tissue. The results are presented in Table 3. In HUVECs, we found 12 differentially expressed genes identified in IA tissue studies that were also present within TADs encompassing IA haplotypes, four of which were reported in multiple studies *(COL1A2, COL3A1*, *COL5A2*, *VCAN*). Using the list of fibroblast TAD genes, we found 11 genes that were also reported as differentially expressed between IA and control tissue. Three of these genes were reported in more than one of the aforementioned studies: *COL1A2*, *COL3A1*, and *VCAN*. Examining the differential expression due to IA rupture, we found 12 genes were within IA-associated TADs of HUVECs. Two genes, *GULP1* and *PLCL1*, were found in multiple studies. Nine genes present in IA-associated TADs of IMR90 fibroblasts were reported to be differentially expressed between unruptured and ruptured IA samples. Again, *GULP1* and *PLCL1* were present in multiple studies, with the addition of *BFSP1*. Combining results for IA vs. control tissue and ruptured vs. unruptured IA tissue, a total of 12% of transcripts within both HUVEC and IMR90 fibroblast TADs were reported as differentially expressed in IA tissue studies.

**Table 3:** Differentially expressed genes in IA tissue present within IA risk associated TADs

	HUVEC TAD		Fib. (IMR90) TAD	
Gene (TAD found in)	*IA* versus *Ctr*	*R* versus *UR*	*IA* versus *Ctr*	*R* versus *UR*
*BFSP1* (rs1132274)				Kleinloog (↓, − 4.0)
				Nakaoka (↓, − 1.8)
*CASD1* (rs42524)		Kurki (↓, − 1.0)		
*CDKN2A* (rs2891168, rs10757278)	Wang (↑, NR)	Nakaoka (↓, − 1.9)	Wang (↑, NR)	Nakaoka (↓, − 1.9)
*CDKN2B* (rs2891168, rs10757278)	Wang (↑, NR)		Wang (↑, NR)	
*COL1A2* (rs42524)	Li (↑, NR)		Li (↑, NR)	
	Wang (↑, NR)		Wang (↑, NR)	
*COL3A1* (rs1800255)	Li (↑, NR)		Li (↑, NR)	
	Wang (↑, NR)		Wang (↑, NR)	
*COL5A2* (rs1800255)	Li (↑, NR)			
	Shi (↑, 3.3)			
	Wang (↑, NR)			
*DSTN* (rs1132274)			Wang (↓, NR)	
*GULP1* (rs1800255)		Kurki (↓, − 2.2)		Kurki (↓, − 2.2)
		Nakaoka (↓, − 1.3)		Nakaoka (↓, − 1.3)
*IL6* (rs1800796)		Kurki (↑, 2.2)		Kurki (↑, 2.2)
*KLHL7* (rs1800796)		Nakaoka (↑, 0.50)		Nakaoka (↑, 0.50)
*MEOX2* (rs4628172)	Wang (↓, NR)		Wang (↓, NR)	
*MOB4* (rs1429412)	Wang (↓, NR)		Wang (↓, NR)	
*PDK4* (rs42524)	Wang (↓, NR)	Kurki (↑, 1.0)		
*PLCL1* (rs700651)		Kleinloog (↓, − 4.0)		Kleinloog (↓, − 4.0)
		Kurki (↓, − 1.4)		Kurki (↓, − 1.4)
*PPP1R9A* (rs42524)	Wang (↓, NR)			
*RRBP1* (rs1132274)			Wang (↑, NR)	
*SGCE* (rs42524)	Wang (↓, NR)	Kurki (↓, − 2.3)		
*SNX5* (rs1132274)			Wang (↑, NR)	
*SOX17* (rs10958409, rs9598506)	Wang (↓, NR)	Nakaoka (↑, 0.88)		
*TFPI* (rs1800255)			Wang (↓, NR)	
*TMEM167A* (rs251124)		Kurki (↑, 1.2)		Kurki (↑, 1.2)
*TOMM7* (rs1800796*)*		Kurki (↓, − 0.86)		Kurki (↓, − 0.86)
*VCAN* (rs251124)	Shi (↑, 2.9)		Shi (↑, 2.9)	
	Wang (↑, NR)		Wang (↑, NR)	
*XRCC4* (rs251124)		Kleinloog (↑, 2.3)		Kleinloog (↑, 2.3)

The log_2_(fold-change) for each gene as reported in the respective study is shown in parentheses. If study did not provide fold-change, NR is reported. Multiple probes corresponded to Nakaoka’s CDKN2A; average value was reported

Ctr., control; Fib., fibroblast; HUVEC, human umbilical vein endothelial cell; IA, intracranial aneurysm; NR, not reported; R, ruptured; TAD, topologically associated domain; UR, unruptured

## Discussion

In this study, we mined publicly-available data from the ENCODE database to gain a better understanding of the nature of genetic risk for IA and how it is related to transcriptional abnormalities observed in human aneurysmal tissue. To do this, we investigated epigenetic landscapes of 16 validated IA risk haplotypes in cells found in the walls of IAs. From ChIP-Seq data, we found evidence that the risk haplotypes defined by the GWAS tag SNPs were enriched for H3K4me1, H3K27ac, and H3K9ac marks, (marks typically associated with enhancer activity [52]), and that these putative regulatory elements were present to a significantly greater degree in the IA-associated risk regions in ECs and fibroblasts rather than other cell types that reside in human IA tissues. While these results are similar to that of our previous work [18], we were able to perform several complementary analyses in this study to gain further insight about epigenetic regulation of gene expression in IA. Indeed, we studied the broader chromatin architecture encompassing the haplotypes that demonstrated H3K4me1/H3K27ac/H3K9ac enrichment, specifically focusing on HiC-defined chromatin loops (i.e. TADs), which are known to facilitate specific enhancer-promoter interactions while limiting others [53]. Bioinformatics analysis of these TADs in HUVECs and IMR90 fibroblasts identified genes plausibly related to IA pathogenesis. A total of 25 of the genes within these TADs have been independently shown to be differentially expressed in human IA tissues. These findings corroborate the idea that genetic modification of enhancers on the risk haplotypes may directly alter gene expression, although the specific enhancer-promoter interactions mediating these effects cannot be ascertained from these types of data. Furthermore, our use of multiple data sources helps to reduce the number of promising target genes through which disease-associated variants may act. We began with those identified in IA-risk associated TADs (164 in HUVECs, 153 in IMR90 fibroblasts) and refined to those genes that also have reported differential expression in IA tissue (20 in HUVECs, 19 in IMR90 cells).

In our recent study [18], we explored the chromatin landscapes associated with these same 16 haplotypes that were reported in a meta-analysis by Alg et al. [19], in order to assess whether there was epigenetic evidence that genetic risk was conferred more predominantly in circulating immune cells or the vascular endothelium. There, we demonstrated that significant enrichment for H3K4me1 and H3K27ac histone peaks was observed exclusively in endothelial cells, compared to peripheral blood leukocytes [18]. Perturbations in endothelial gene expression are thought to be among the first pathobiological vascular responses that precede IA formation [54–57]. Thus, these results had indicated that genetic risk for IA may be conferred through the vascular wall rather than circulating immune cells, which prompted the current study’s investigation of additional cell types found in arterial tissues, as well as broader chromatin architecture that subsumes the risk haplotypes. The current study corroborates our earlier finding that HUVECs showed significant enrichment for H3K4me1 (present in 13/16 LD blocks, *p* = 0.00025) and H3K27ac (present in 9/16 LD blocks, *p* = 0.0031) ChIP-Seq peaks, since the same list of 16 LD blocks and same ChIP-Seq dataset was used. In that study, the sites marked by H3K4me1 and H3K27ac in HUVECs had also demonstrated abundant transcription factor binding sites and CTCF binding sites, further signifying that these regions were associated with functional enhancer activity.

In the current study, we were able to perform additional analyses using H3K9ac ChIPseq data, another indicator of functionally active chromatin [52, 58, 59]. We found that this mark demonstrated significant enrichment (*p* = 0.014) and was present in 7 of the LD blocks for HUVECs. We also investigated ChIP-Seq data from HAECs, an endothelial cell line that may be more biologically relevant to the arterial vasculature where IAs form than venous cells from the umbilical cord. However, we were only able to show significant enrichment of H3K27ac marks (present in 10/16 LD blocks, *p* = 0.01), as ChIP-Seq data was unavailable for H3K4me1 and H3K9ac. Taken together, these data yet again implicate ECs, which have been widely recognized as a key player in IA natural history [55, 56, 60], as a main cell type to be most likely affected by genetic risk for IA.

In addition to ECs, the other cell type we investigated that had significant enrichment for the ChIP-Seq peaks of interest were fibroblasts. While not extensively studied in IA, fibroblasts are known to coordinate the production of collagen (particularly Type I), as well as produce and organize extracellular matrix, where they resize and carry out adherent and contractile abilities [61]. Additionally, they can produce matrix metalloproteinases, which facilitate vessel wall destruction and remodeling during IA formation [62, 63]. In our data, skin fibroblasts showed significant enrichment in H3K4me1 (present in 7/16 LD blocks, *p* = 0.0091), H3K27ac (present in 9/16 LD blocks, *p* = 0.0027), and H3K9ac marks (present in 6/16 LD blocks, *p* = 0.010), while lung fibroblasts only showed enrichment of H3K27ac (present in 8/16 LD blocks, *p* = 0.0079). Therefore, it appears that, in addition to ECs, fibroblasts are another cellular component of the vascular wall where the biological effects of genetic variants may confer risk for the development of IA.

To identify genes potentially affected by enhancers in the LD blocks investigated in this study, we examined the TADs (regions of the genome that form interactions and thereby affect gene expression together) encompassing the 16 LD blocks of interest. For ECs, ontology annotations associated with genes within the TADs reflected extracellular matrix structure and enzymatic activity. Furthermore, disease and function annotation in IPA showed significant enrichment of Loeys-Dietz syndrome, Ehlers-Danlos syndrome, and vascular lesion. The bioinformatics results broadly reflect the connective tissue weakening that is central to the pathogenesis and progression of IA. This is further reflected in hub genes of significant networks, including multiple forms of collagen and VCAN, or versican, a proteoglycan that is a major component of the extracellular matrix of vessels.

In associated TADs in fibroblasts, there was a large degree of overlap between genes found within HUVECs. Therefore, it is not surprising that bioinformatics performed on this group of genes also reflected phosphorylase activity. Additionally, IPA networks of gene interactions reflected many hub molecules identified in our analysis of ECs, i.e. collagens and VCAN. However, they also reflected immune responses, with hubs at Akt, ERK1/2, and NF-kB (all transcription factors) in addition to a large node at *IL6*. NF-kB is an important mediator of gene expression in IA formation that can regulate the expression of may interleukins, cytokines, and chemokines known to play key roles in IA natural history.

It is important to recognize that enhancer function cannot be determined on the basis of chromatin marks alone. Therefore, to further assess if genes located in TADs subsuming the IA-associated LD blocks in ECs and fibroblasts were, indeed, differentially expressed in IA tissue, we compared our list of potential targets to differentially expressed genes reported in numerous studies (see Table 3). In studies that compared control vascular tissue (primarily superior temporal artery tissue), HUVEC and fibroblast TAD genes included structural collagens, *COL1A2* and *COL3A1*, which were found to be increased in IA tissue. The inclusion of *VCAN*, which also was increased in IA tissue, further reflects the role of dysregulated extracellular matrix components, as it plays a role in cell adhesion, proliferation, migration, and angiogenesis [64]. Differential expression of *CDKN2A/B*, a gene that is involved in regulating EC proliferation and repair in an effort to prevent vascular injury [12], was also present in the HUVEC and fibroblast TAD genes, as was *MEOX2*, a transcription factor that activates the expression of *CDKN2A* [65]. Multiple GWAS show that an exonic SNP in *CDKN2* (9p213) is highly associated with IA. However, our results may indicate that this gene could also be regulated by aberrant enhancer activity. Ultimately, these data support the idea that genetic risk is exerted predominantly on extracellular matrix processes carried out by vascular ECs and fibroblasts.

Studies that compared ruptured IA tissue to unruptured IA tissue shared many of these same genes that were also present in HUVEC and fibroblast TADs. There were several genes that were uniquely differentially expressed when unruptured IAs were compared to ruptured ones. *KLHL7*, which may participate in protein degradation, is overexpressed in ruptured IA cases. *IL6*, also increased in ruptured cases, is a proinflammatory cytokine that has long been recognized in IA pathology [66]. *IL6* promoter polymorphisms, such as rs1800796, are also associated with IA and other inflammatory diseases [67]. Consequently, we suspect that in addition to extracellular matrix processes, genetic risk may also affect inflammatory responses in the vessel wall. Interestingly, the effect of SNPs on expression of *IL6* may be similar to that on *CDKN2*. Both regions containing prominent histone marks within the gene, which may function as so-called “exonic enhancers” [68]. In such a case, the genetic variant could operate through the gene sequence function, the enhancer function, or both to lead to differential expression of the gene. Taken together, our results show strong support that the studied regions contain active enhancers, in which disruption by SNPs causes aberrant changes in gene expression. However, experimental studies empirically demonstrating the effect of altered enhancer activity of IA-associated variants is still needed.

Our study has several limitations. First, we focused on SNPs identified in one meta-analysis. While this meta-analysis was comprehensive and the SNPs identified were found in at least two studies that analyzed a large volume of cases in controlled populations, a larger study in the future including SNPs from other works would be beneficial. Second, we generalized our results from HUVECs and SMCs to IA samples, though these cell types may be different in arterial aneurysm tissue. When possible, we used cell types that more reflected those in IA walls, such as HAECs instead of HUVECs. However, in many cases we were limited by data availability, i.e. there was not as much Hi-C data available for our TAD analysis as we would have liked. For example, HUVECs had widely-available datasets, but fibroblasts did not, and thus we resorted to using data from IMR90 cells instead of skin fibroblasts for TAD analysis. We are currently planning experimental validations of these findings in cell lines more similar to IA-resident cells. Lastly, we recognize that gene expression studies in IA tissue report differential expression that is aggregated from the many cell types that are present in the aneurysm. Single-cell studies would be needed to verify differential expression of genes within IA-associated TADs from one cell type vs. another.

## Conclusions

We analyzed H3K4me1, H3K27ac, and H3K9ac histone marks in 16 IA-risk regions, reporting that genetic risk for IA is conferred through ECs and fibroblasts. Our findings provide evidence that genetic variants known to be associated with IA risk act on endothelial cells and fibroblasts, rather than immune cells or smooth muscle cells. Based on analysis of genes in overlapping TADs, genetic risk of IA may affect regulation of genes involved in extracellular matrix integrity in ECs and fibroblasts. There is strong circumstantial evidence that this may be mediated through altered enhancer function, as multiple genes in TADs encompassing the enhancer marks have also been shown to be differentially expressed in the IA tissue.

## Supplementary Information

**Additional file 1: Fig. S1**. Visualization of the topologically associated domains (TADs) of four example SNPs in fibroblasts (IMR90 cells). Tiff image. **Fig. S2**. Additional IPA networks of transcripts in TADs encompassing predicted enhancers in HUVECs and fibroblasts. Tiff image. **Table S1**. Cistrome datasets for histone mark analysis. **Table S2**. Conversion of IA associated linkage disequilibrium blocks to hg38. **Table S3**. Genes encompassed within TADs surrounding IA-risk associated haplotypes. **Table S4**. gProfiler GO ontologies of genes within IA associated, histone marked TADs. **Table S5**. IPA diseases and biological functions of genes within IA associated, histone marked TADs. **Table S6**. IPA Networks of genes within IA associated, histone marked TADs. **Table S7**. IA tissue differential expression studies.

## Data Availability

The GEO/ENCODE IDs for the datasets obtained through Cistrome database [http://cistrome.org/db/#/] are as follows: NK cell H3K27ac – GSM999008, H3K4me1 – GSM999007; CD4 + T cell H3K27ac – GSM1220560, H3K4me1 – GSM1220567, H3K9ac – GSM543004; CD8 + T cell H3K27ac – GSM1102781, H3K4me1 – GSM1220569, H3K9ac – GSM613813; SMC H3K27ac – ENCSR210ZPC_2, H3K4me1 – ENCSR130IMV_2, H3K9ac – ENCSR540UZV_2; GM12878 cell H3K27ac – GSM733771, H3K4me1 – GSM733772, H3K9ac – GSM733677; HUVEC H3K27ac – GSM733691, H3K4me1 – GSM733690, H3K9ac – GSM733735, H3K9me3 – GSM1003517; ung fibroblast H3K27ac – GSM733646, H3K4me1 – GSM733649, H3K9ac – GSM733652, H3K9me3 – GSM733695; Skin fibroblast H3K27ac – GSM733662, H3K4me1 – GSM1003526, H3K9ac – GSM733709, H3K9me3 – GSM733744; Monocyte H3K27ac – GSM1003559, H3K4me1 – GSM1003535, H3K9ac – GSM1003515; Neutrophil H3K27ac – GSM2527660, H3K4me1 – GSM2534489; HAEC H3K27ac – GSM2394402; Macrophage H3K27ac – GSM785500, H3K4me1 – GSM785498; The GEO ID for the dataset used in the 3D genome browser [http://3dgenome.fsm.northwestern.edu/view.php] is GSE63525.

## Abbreviations

DEG: Differentially expressed gene
EC: Endothelial cell
GO: Gene ontology
GWAS: Genome-wide association study
HAEC: Human aortic endothelial cell
HUVEC: Human umbilical vein endothelial cell
IA: Intracranial aneurysm
IPA: Ingenuity pathway analysis
LD: Linkage disequilibrium
NK: Natural killer
OR: Odds ratio
PBMC: Peripheral blood mononuclear cell
PNL: Polymorphonuclear leukocyte
SMC: Smooth muscle cell
SNP: Single nucleotide polymorphism
TAD: Topologically associated domain
