# Supplementary material for: Epigenetic landscapes of intracranial aneurysm risk haplotypes implicate enhancer function of endothelial cells and fibroblasts in dysregulated gene expression
Source: BMC Med Genomics. 2021 Jun 16;14:162. doi: 10.1186/s12920-021-01007-9 (PMC8210394; doi:10.1186/s12920-021-01007-9)
Supplement: Supplementary file 1 — Additional file 1: Fig. S1. Visualization of the topologically associated domains (TADs) of four example SNPs in fibroblasts (IMR90 cells). Tiff image. Fig. S2. Additional IPA networks of transcripts in TADs encompassing predicted enhancers in HUVECs and fibroblasts. Tiff image. Table S1. Cistrome datasets for histone mark analysis. Table S2. Conversion of IA associated linkage disequilibrium blocks to hg38. Table S3. Genes encompassed within TADs surrounding IA-risk associated haplotypes. Table S4. gProfiler GO ontologies of genes within IA associated, histone marked TADs. Table S5. IPA diseases and biological functions of genes within IA associated, histone marked TADs. Table S6. IPA Networks of genes within IA associated, histone marked TADs. Table S7. IA tissue differential expression studies. [file 12920_2021_1007_MOESM1_ESM.docx]

**Supplemental Information for:**

**Epigenetic Landscapes of Intracranial Aneurysm Risk Haplotypes Implicate Enhancer Function of Endothelial Cells and Fibroblasts in Dysregulated Gene Expression**

Kerry E. Poppenberg PhD^1,2^, Haley R. Zebraski BS^1,3^, Naval Avasthi BS^1,3^, Muhammad Waqas MBBS^1,2^, Adnan H. Siddiqui MD PhD^1,2^, James N. Jarvis MD^4^, *Vincent M. Tutino PhD^1,2,3,5,6^

**Affiliations**: ^1^Canon Stroke and Vascular Research Center; ^2^Department of Neurosurgery, ^3^Department of Biomedical Engineering, ^4^Department of Pediatrics, ^5^Department of Pathology and Anatomical Sciences, ^6^Department of Mechanical and Aerospace Engineering, University at Buffalo, Buffalo, NY, USA

**Email Addresses**: kerrypop@buffalo.edu, haleyzeb@buffalo.edu, navalava@buffalo.edu, mwaqas@ubns.com, asiddiqui@ubns.com, jamesjar@buffalo.edu, vincentt@buffalo.edu

***Correspondence**:

Vincent Tutino, PhD

Canon Stroke and Vascular Research Center

Clinical and Translational Research Center

875 Ellicott Street

Buffalo, NY 14214 USA

E-mail: vincentt@buffalo.edu; Phone: (716) 829-5400

Fax: (716) 854-1850

**Supplemental Figures**

**Figure S1. Visualization of the topologically associated domains (TADs) of four example SNPs in fibroblasts (IMR90 cells).** Haplotype outlined in red. TAD outlined in black. Genes reported as differentially expressed in IA tissue studies are highlighted in red (increased expression) or green (decreased expression). Border indicates identified in IA vs control; fill indicates identified in ruptured vs unruptured IAs. **A).** HiC data surrounding rs1429412 shows a large TAD in fibroblasts.

**B).** Data from HiC analysis shows a large TAD surrounding rs1800796. **C).** HiC data shows smaller TAD surrounding rs10757278. **D).** HiC map showing TAD encompassing rs6538595 does not contain any genes found to be differentially expressed in IA tissue studies.


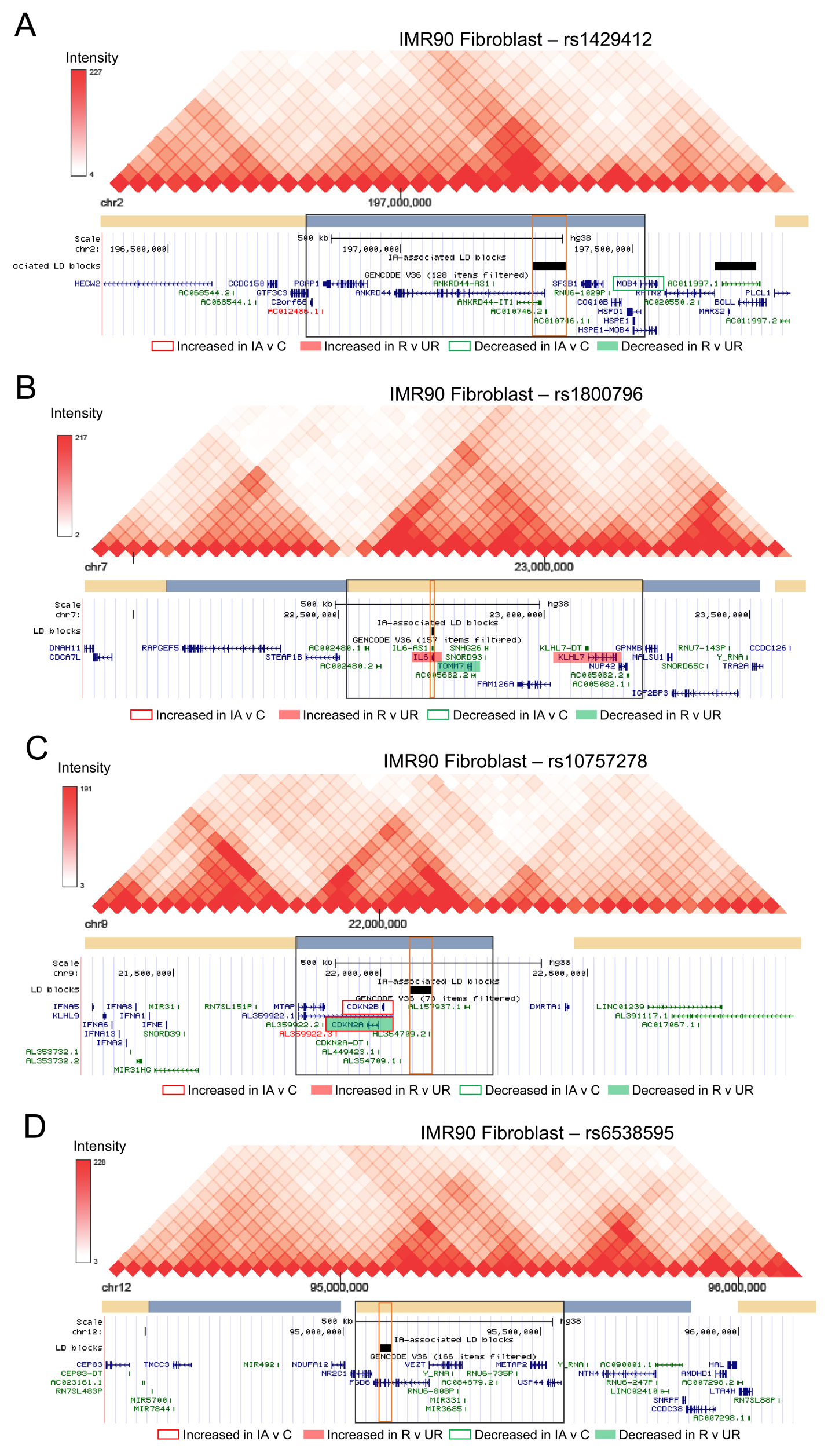


**Figure S2. Additional IPA networks of transcripts in TADs encompassing predicted enhancers in HUVECs and fibroblasts.** Transcripts present in TADs are in grey and lines indicate interactions (solid=direct, dashed=indirect). Genes reported as differentially expressed in IA tissue studies are highlighted in red (increased expression) or green (decreased expression). Border indicates identified in IA vs control; fill indicates identified in ruptured vs unruptured IAs. **A).** In HUVECs, a network with p-score=30 demonstrated interaction hubs at STAT5A/B, TP53, and ELAVL1. **B).** The second network for fibroblasts had a p-score=25 and, similar to the first HUVEC network, had hubs of interactions at MAPK, CDKN2A/B, ERK1/2, and collagens. **C).** The third network of transcripts for fibroblasts had a p-score=25 and had interactions around TGFB and ESR2.


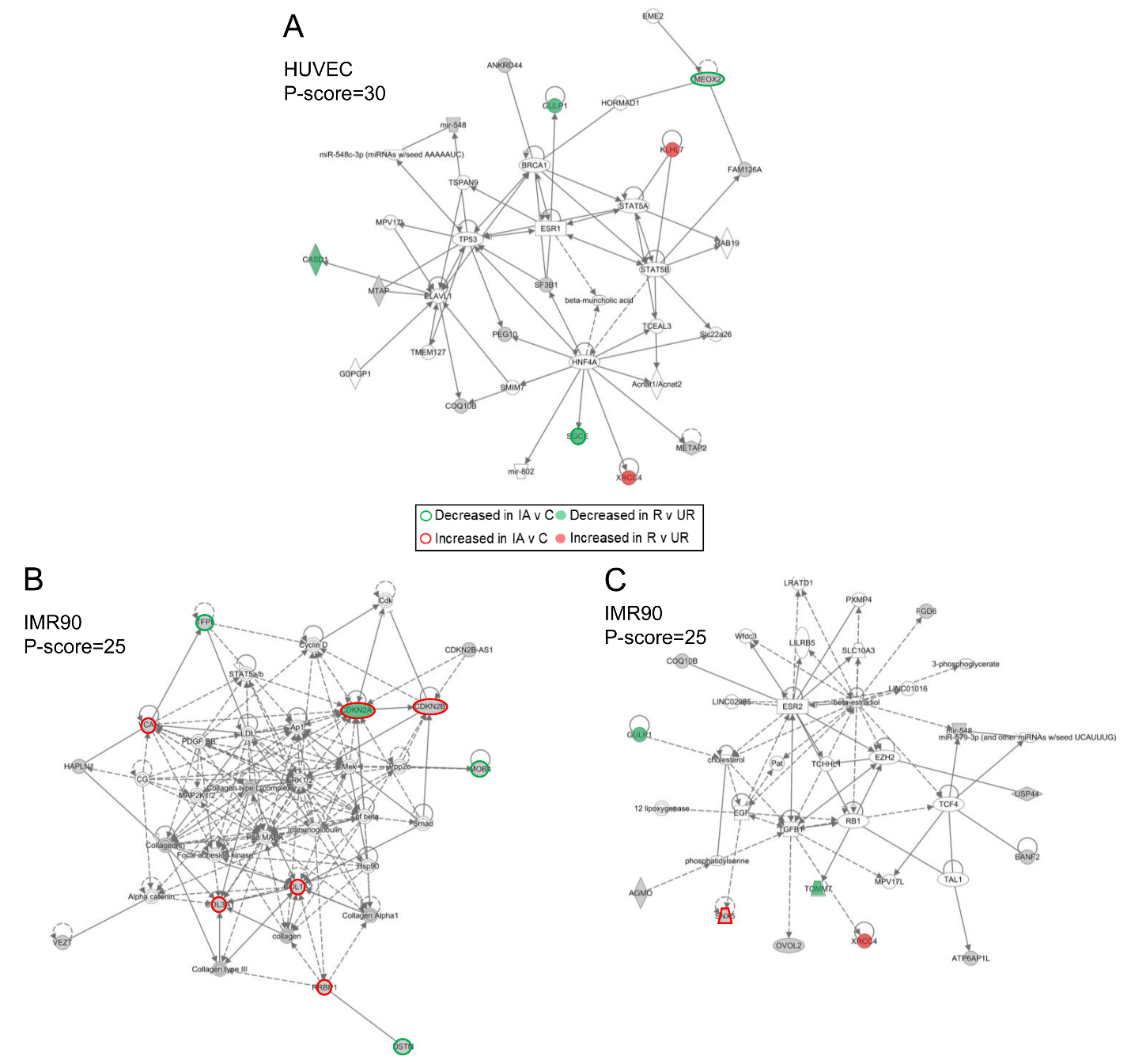


**Table S1: Cistrome datasets for histone mark analysis.***

| **Year** | **1^st^ Author** | **Journal** | **Source** | **Factor** | **GEO/ENCODE ID** |
| --- | --- | --- | --- | --- | --- |
| 2014 | Andersson, R. | Nature | NK (Blood) | H3K27ac | GSM999008 |
|  |  |  |  | H3K4me1 | GSM999007 |
| 2010 | Bernstein, B.E. | Nat. Biotechnol. | T Lym. (Blood - CD4+) | H3K27ac | GSM1220560 |
|  |  |  |  | H3K4me1 | GSM1220567 |
|  |  |  |  | H3K9ac | GSM543004 |
|  |  |  | T Lym. (Blood - CD8+) | H3K27ac | GSM1102781 |
|  |  |  |  | H3K4me1 | GSM1220569 |
|  |  |  |  | H3K9ac | GSM613813 |
| 2018 | Davis, C.A. | Nucleic Acids Res. | SMC | H3K27ac | ENCSR210ZPC_2 |
|  |  |  |  | H3K4me1 | ENCSR130IMV_2 |
|  |  |  |  | H3K9ac | ENCSR540UZV_2 |
| 2012 | ENCODE Project Consortium | Nature | GM12878; B Lym.; Blood | H3K27ac | GSM733771 |
|  |  |  |  | H3K4me1 | GSM733772 |
|  |  |  |  | H3K9ac | GSM733677 |
|  |  |  | HUVEC | H3K27ac | GSM733691 |
|  |  |  |  | H3K4me1 | GSM733690 |
|  |  |  |  | H3K9ac | GSM733735 |
|  |  |  |  | H3K9me3 | GSM1003517 |
|  |  |  | Fib. (Lung) | H3K27ac | GSM733646 |
|  |  |  |  | H3K4me1 | GSM733649 |
|  |  |  |  | H3K9ac | GSM733652 |
|  |  |  |  | H3K9me3 | GSM733695 |
|  |  |  | Fib. (Skin) | H3K27ac | GSM733662 |
|  |  |  |  | H3K4me1 | GSM1003526 |
|  |  |  |  | H3K9ac | GSM733709 |
|  |  |  |  | H3K9me3 | GSM733744 |
|  |  |  | Mono. | H3K27ac | GSM1003559 |
|  |  |  |  | H3K4me1 | GSM1003535 |
|  |  |  |  | H3K9ac | GSM1003515 |
|  |  |  | PNL; Blood | H3K27ac | GSM2527660 |
|  |  |  |  | H3K4me1 | GSM2534489 |
| 2017 | Hogan, N.T. | Elife | HAEC | H3K27ac | GSM2394402 |
| 2012 | Pham, T.H. | Blood | M0 | H3K27ac | GSM785500 |
|  |  |  |  | H3K4me1 | GSM785498 |

* Abbreviations: fib.=fibroblast, HAEC=human aortic endothelial cell, HUVEC=human umbilical vein endothelial cell, lym.=lymphocyte, M0=macrophage, mono.=monocyte, NK=natural killer cell, PNL=polymorphonuclear leukocyte, SMC=smooth muscle cell.

**Table S2: Conversion of IA associated linkage disequilibrium blocks to hg38.***

| **SNP** | **LD Block (hg19)** | **LD Block (hg38)** | **Length (bp)** |
| --- | --- | --- | --- |
| rs3767137 | chr1:22160723–22168310 | chr1:21834230–21841817 | 7587 |
| rs1800255 | chr2:189841613–189867882 | chr2:188976887–189003156 | 26269 |
| rs1429412 | chr2:198148191–198223121 | chr2:197283467–197358397 | 74930 |
| rs700651 | chr2:198541398–198631714 | chr2:197676674–197766990 | 90316 |
| rs6841581 | chr4:148365339–148414651 | chr4:147444187–147493499 | 49312 |
| rs251124 | chr5:82805424–82826254 | chr5:83509605–83530435 | 20830 |
| rs4628172 | chr7:15493884–15506529 | chr7:15454259–15466904 | 12645 |
| rs1800796 | chr7:22766246–22771738 | chr7:22726627–22732119 | 5492 |
| rs42524 | chr7:94043239–94049356 | chr7:94413927–94420044 | 6117 |
| rs10958409 | chr8:55309731–55328116 | chr8:54397171–54415556 | 18385 |
| rs9298506 | chr8:55421614–55462324 | chr8:54509054–54549764 | 40710 |
| rs2891168 | chr9:22072264–22125503 | chr9:22072265–22125504 | 53239 |
| rs10757278 | chr9:22077085–22125503 | chr9:22077086–22125504 | 48418 |
| rs6538595 | chr12:95489131–95516843 | chr12:95095355–95123067 | 27712 |
| rs4934 | chr14:95078677–95080803 | chr14:94612340–94614466 | 2126 |
| rs1132274 | chr20:17594030–17600114 | chr20:17613385–17619469 | 6084 |

* Abbreviations: hg=human genome, IA=intracranial aneurysm, LD=linkage disequilibrium, rs=reference SNP cluster ID, SNP=single nucleotide polymorphism, chr=chromosome.

**Table S3: Genes encompassed within TADs surrounding IA-risk associated haplotypes.***

| **SNP** | **LD Block** | **Genes within TAD** |  |
| --- | --- | --- | --- |
| **HUVEC** | | |  |
| rs1800255 | chr2:188976887-189003156 | AC092598.1, AC133106.1, **COL3A1**, **COL5A2**, DIRC1, **GULP1**, KRT18P19, MIR1245A, MIR1245B, MIR3129, MIR3606 |  |
| rs1429412 | chr2:197283467-197358397 | AC010746.1, AC010746.2, AC013264.1, AC017035.1, AC02050.1, ANKRD44, ANKRD44-IT1, ATP5MC2P3, C2ORF66, COQ10B, HNRNPA3P15, HSPD1, HSPE1, HSPE1-MOB4, **MOB4**, NPM1P46, PGAP, RNU6-1029P, SF3B1 |  |
| rs700651 | chr2:197676674-197766990 | AC011997.1, BOLL, **PLCL1** |  |
| rs6841581 | chr4:147444187-147493499 | AC010683.1, AC093908.1, AC097450.1, EDNRA, GTF2F2P1, LINC02507, MIR548G, PRMT5P1 |  |
| rs251124 | chr5:83509605-83530435 | AC008885.1, AC008885.2, AC026782.1, AC026782.2, AC027338.1, AC027338.2, AC094085.1, AC104118.1, AC108174.1, ATP6AP1L, COQ10BP2, FTH1P9, HAPLN1, LINC01338, MIR3977, RPL5P16, RPS23, SCARNA18, ST13P12, **TMEM167A**, VCAN, VCAN-AS1, **XRCC4** |  |
| rs4628172 | chr7:15454259-15466904 | AC005550.1, AC005550.2, AC006041.1, AC006041.2, AC006150.1, AC006458.1, AGMO, CRPPA, DGKB, GTF3AP5, LINC02587, **MEOX2**, RPL36AP26 |  |
| rs1800796 | chr7:22726627-22732119 | AC002480.1, AC002480.2, AC005082.1, AC005682.1, AC005682.2, AC073072.1, AC073072.2, AK3P3, FAM126A, **IL6**, **KLHL7**, KLHL7-DT, MTCYBP42, NUP42, RPL12P10, SNHG26, SNORD93, **TOMM7** |  |
| rs42524 | chr7:94413927-94420044 | AC002429.1, AC002429.2, AC002451.1, AC002451.2, AC004012.1, AC004022.1, AC004022.2, AC005021.1, ARF1P1, ASB4, ATP5PBP2, **CASD1**, **COL1A2**, GRPEL2P3, HINT1P2, **PDK4**, PEG10, PON1, PON2, PON3, **PPP1R9A**, RN7SKP129, RNU4-16P, RNU6-1328P, RNU6-956P |  |
| rs10958409 | chr8:54397171-54415556 | AC027250.1, AC027250.2, AC044836.1, AC060764.1, AC091076.1, RN7SL250P, RNU105C, SEC11B, **SOX17**, TRMT112P7 |  |
| rs9298506 | chr8:54509054-54549764 | AC027250.1, AC027250.2, AC044836.1, AC060764.1, AC091076.1, RN7SL250P, RNU105C, SEC11B, **SOX17**, TRMT112P7 |  |
| rs2891168 | chr9:22072265-22125504 | AL157937.1, AL354709.1, AL354709.2, AL359922.1, AL359922.2, AL359922.3, AL449423.1, **CDKN2A**, CDKN2A-DT, **CDKN2B**, CDKN2B-AS1, DMRTA1, ERVFRD-3, MTAP, TUBB8P1, UBA52P6 |  |
| rs10757278 | chr9:22077086-22125504 | AL157937.1, AL354709.1, AL354709.2, AL359922.1, AL359922.2, AL359922.3, AL449423.1, **CDKN2A**, CDKN2A-DT, **CDKN2B**, CDKN2B-AS1, DMRTA1, ERVFRD-3, MTAP, TUBB8P1, UBA52P6 |  |
| rs6538595 | chr12:95095355-95123067 | AC018475.1, AC084879.1, AC084879.2, AC126615.1, AC126615.2, CBX3P5, FGD6, METAP2, MIR331, MIR3685, PGAM1P5, RNU6-735P, RNU6-808P, USP44, VEZT, Y_RNA |  |
| **Fibroblast (IMR90)** | | | |
| rs1800255 | chr2:188976887-189003156 | AC092598.1, AC104131.1, **COL3A1**, DIRC1, **GULP1**, LINC01090, MIR1245A, MIR1245B, MIR3606, MIR561, RNA5SP114, ST13P2, **TFPI** |  |
| rs1429412 | chr2:197283467-197358397 | AC010746.1, AC010746.2, AC013264.1, AC017035.1, AC020550.1, ANKRD44, ANKRD44-IT1, ATP5MC2P3, C2ORF66, COQ10B, HNRNPA3P15, HSPD1, HSPE1, HSPE1-MOB4, **MOB4**, NPM1P46, PGAP, RNU6-1029P, SF3B1 |  |
| rs700651 | chr2:197676674-197766990 | AC011997.2, BOLL, **PLCL1** |  |
| rs6841581 | chr4:147444187-147493499 | AC010683.1, AC093908.1, AC097450.1, EDNRA, GTF2F2P1, LINC02507, MIR548G, PRMT5P1 |  |
| rs251124 | chr5:83509605-83530435 | AC008885.1, AC008885.2, AC026782.1, AC026782.2, AC027338.1, AC027338.2, AC094085.1, AC104118.1, AC108174.1, ATP6AP1L, COQ10BP2, FTH1P9, HAPLN1, LINC01338, MIR3977, RPL5P16, RPS23, SCARNA18, ST13P12, **TMEM167A**, **VCAN**, VCAN-AS1, **XRCC4** |  |
| rs4628172 | chr7:15454259-15466904 | AC005550.1, AC005550.2, AC006041.1, AC006041.2, AGMO, LINC02587, **MEOX2**, RPL36AP26 |  |
| rs1800796 | chr7:22726627-22732119 | AC002480.1, AC002480.2, AC005082.1, AC005082.2, AC005682.1, AC005682.2, AC073072.1, AC073072.2, AK3P3, FAM126A, **IL6**, **KLHL7**, KLHL7-DT, MTCYBP42, NUP42, RPL12P10, SNHG26, SNORD93, **TOMM7** |  |
| rs42524 | chr7:94413927-94420044 | AC002074.1, AC002074.2, **COL1A2** |  |
| rs2891168 | chr9:22072265-22125504 | AL157937.1, AL354709.1, AL354709.2, AL359922.1, AL359922.2, AL449423.1, **CDKN2A**, CDKN2A-DT, **CDKN2B**, CDKN2B-AS1, ERVFRD-3, MTAP, TUBB8P1, UBA52P6 |  |
| rs10757278 | chr9:22077086-22125504 | AL157937.1, AL354709.1, AL354709.2, AL359922.1, AL359922.2, AL449423.1, **CDKN2A**, CDKN2A-DT, **CDKN2B**, CDKN2B-AS1, ERVFRD-3, MTAP, TUBB8P1, UBA52P6 |  |
| rs6538595 | chr12:95095355-95123067 | AC018475.1, AC084879.1, AC084879.2, AC126615.1, CBX3P5, FGD6, METAP2, MIR331, MIR3685, NR2C1, PGAM1P5, RNU6-735P, RNU6-808P, USP44, VEZT, Y_RNA |  |
| rs1132274 | chr20:17613385-17619469 | AL035045.1, AL049646.1, AL049646.3, AL050321.1, AL132765.1, AL132765.2, AL160411.1, BANF2, **BFSP1**, **DSTN**, KAT14, MGME1, OVOL2, PET117, PTMAP3, RN7SKP69, RN7SKP74, RN7SL14P, RNU2-56P, RNU6-192P, RNU7-137P, RPL15P1, RPS27AP2, **RRBP1**, SNORD17, **SNX5**, Y_RNA, ZNF133 |  |

*Genes in bold were reported as differentially expressed in one or more of the IA tissue studies. Abbreviations: IA=intracranial aneurysm, rs=reference SNP cluster ID, SNP=single nucleotide polymorphism, HUVEC=human umbilical vein endothelial cell, TAD=topologically associated domain, LD=linkage disequilibrium, chr=chromosome.

**Table S4: gProfiler GO ontologies of genes within IA associated, histone marked TADs.***

| **GO** | **GO ID** | **GO Name** | **q-value** | **Genes** |
| --- | --- | --- | --- | --- |
| **HUVEC** | | | | |
| MF | GO:0046573 | lactonohydrolase activity | 0.00001 | PON1, PON3, PON2 |
| MF | GO:0102007 | acyl-L-homoserine-lactone lactonohydrolase activity | 0.00001 | PON1, PON3, PON2 |
| MF | GO:0004064 | arylesterase activity | 0.00016 | PON1, PON3, PON2 |
| MF | GO:0004063 | aryldialkylphosphatase activity | 0.00323 | PON1, PON3 |
| MF | GO:0017061 | S-methyl-5-thioadenosine phosphorylase activity | 0.01930 | MTAP, AL359922.1 |
| MF | GO:0004731 | purine-nucleoside phosphorylase activity | 0.03212 | MTAP, AL359922.1 |
| MF | GO:0005201 | extracellular matrix structural constituent | 0.03605 | COL3A1, COL5A2, VCAN, HAPLN1, COL1A2 |
| BP | GO:0019372 | lipoxygenase pathway | 0.01775 | PON1, PON3, PON2 |
| CC | GO:0005583 | fibrillar collagen trimer | 0.00086 | COL3A1, COL5A2, COL1A2 |
| CC | GO:0098643 | banded collagen fibril | 0.00086 | COL3A1, COL5A2, COL1A2 |
| CC | GO:0098644 | complex of collagen trimers | 0.00514 | COL3A1, COL5A2, COL1A2 |
| **Fibroblast (IMR90)** | | | | |
| MF | GO:0017061 | S-methyl-5-thioadenosine phosphorylase activity | 0.01426 | MTAP, AL359922.1 |
| MF | GO:0004731 | purine-nucleoside phosphorylase activity | 0.02373 | MTAP, AL359922.1 |

* Abbreviations: IA=intracranial aneurysm, HUVEC=human umbilical vein endothelial cell, TAD=topologically associated domain, BP=biological process, CC=cellular component, GO=gene ontology, MF=molecular function.

**Table S5: IPA diseases and biological functions of genes within IA associated, histone marked TADs.***

| **Categories** | **Diseases or Functions Annotation** | **q-value** | **Molecules** | |  |
| --- | --- | --- | --- | --- | --- |
| **HUVEC** | | | | | |
| Endocrine System Disorders, Gastrointestinal Disease, Metabolic Disease, Organismal Injury and Abnormalities | Diabetic complication | 0.00757 | AGMO, CDKN2A, COL1A2, COL3A1, COL5A2, EDNRA, HAPLN1, IL6, PDK4 |  |  |
| Cancer, Organismal Injury and Abnormalities, Reproductive System Disease | Female genital tract serous carcinoma | 0.00771 | CDKN2A, COL3A1, EDNRA, IL6, MEOX2, PDK4, PLCL1, VCAN |  |  |
| Developmental Disorder, Hereditary Disorder, Organismal Injury and Abnormalities, Skeletal and Muscular Disorders | Progressive muscular dystrophy | 0.00187 | COL1A2, COL3A1, COL5A2, CRPPA, mir-331, mir-548, PDK4 |  |  |
| Cardiovascular Disease | Vascular lesion | 0.00757 | COL3A1, COL5A2, EDNRA, IL6, PON1, PON3, VCAN |  |  |
| Cancer, Organismal Injury and Abnormalities, Reproductive System Disease | Uterine serous papillary cancer | 0.00757 | CDKN2A, COL3A1, EDNRA, IL6, MEOX2, PLCL1 |  |  |
| Connective Tissue Disorders, Developmental Disorder, Hereditary Disorder, Organismal Injury and Abnormalities, Skeletal and Muscular Disorders | Autosomal dominant skeletal dysplasia | 0.00187 | COL1A2, COL3A1, COL5A2, EDNRA, MTAP |  |  |
| Developmental Disorder, Hereditary Disorder, Organismal Injury and Abnormalities, Skeletal and Muscular Disorders | Duchenne muscular dystrophy | 0.00642 | COL1A2, COL3A1, COL5A2, mir-548, PDK4 |  |  |
| Cell Morphology | Morphology of antigen presenting cells | 0.00752 | CDKN2A, CDKN2B, IL6, PON1, PON2 |  |  |
| Organismal Injury and Abnormalities, Tissue Morphology | Area of lesion | 0.00642 | CDKN2A, IL6, PON1, PON3 |  |  |
| Cardiovascular Disease, Connective Tissue Disorders, Developmental Disorder, Hereditary Disorder, Organismal Injury and Abnormalities, Skeletal and Muscular Disorders | Loeys-Dietz syndrome | 0.00474 | COL3A1, COL5A2, VCAN |  |  |
| Cell Morphology, Inflammatory Response | Size of phagocytes | 0.00639 | IL6, PON1, PON2 |  |  |
| Cell Morphology, Hematological System Development and Function | Size of myeloid cells | 0.00639 | IL6, PON1, PON2 |  |  |
| Connective Tissue Disorders, Dermatological Diseases and Conditions, Developmental Disorder, Hereditary Disorder, Metabolic Disease, Organismal Injury and Abnormalities, Skeletal and Muscular Disorders | Ehlers-Danlos syndrome | 0.00861 | COL1A2, COL3A1, COL5A2 |  |  |
| **Fibroblast (IMR90) –** *No disease and biological functions* | | | |  |  |
| - | - | - | - |  |  |

* Abbreviations: IA=intracranial aneurysm, IPA=Ingenuity pathway analysis, HUVEC=human umbilical vein endothelial cell, TAD=topologically associated domain.

**Table S6: IPA Networks of genes within IA associated, histone marked TADs.***

| **Network** | **Molecules in Network** | **P-Score** | **Focus Molecules** | **Top Diseases and Functions** |
| --- | --- | --- | --- | --- |
| **HUVEC** | | | | |
| 1 | Alpha catenin, Cdk, CDKN2A, CDKN2B, CDKN2B-AS1, CG, COL1A2, COL3A1, COL5A2, collagen, Collagen type I (complex), Collagen(s), Cyclin D, ERK1/2, Growth hormone, HAPLN1, HSPE1, LDL, MAP2K1/2, Mek, MIRLET7, MOB4, PDGF BB, PDK4, PLCL1, PON1, Ppp2c, Rsk, Smad, Smad2/3, SOX17, STAT5a/b, Tgf beta, VCAN, VEZT | 33 | 15 | Cardiovascular Disease, Connective Tissue Disorders, Developmental Disorder |
| 2 | Acnat1/Acnat2, ANKRD44, beta-muricholic acid, BRCA1, CASD1, COQ10B, ELAVL1, EME2, ESR1, FAM126A, GDPGP1, GULP1, HNF4A, HORMAD1, KLHL7, MEOX2, METAP2, mir-548, miR-548c-3p (miRNAs w/seed AAAAAUC), mir-802, MPV17L, MTAP, PEG10, RAB19, SF3B1, SGCE, Slc22a26, SMIM7, STAT5A, STAT5B, TCEAL3, TMEM127, TP53, TSPAN9, XRCC4 | 30 | 14 | Cellular Assembly and Organization, Organismal Injury and Abnormalities, Reproductive System Disease |
| **Fibroblast (IMR90)** | | | | |
| 1 | ADCY, Akt, caspase, CD3, cytochrome C, EDNRA, ERK, FAM126A, Hdac, hemoglobin, Histone h3, Hsp70, HSPD1, HSPE1, IgG, IL6, Insulin, Interferon alpha, Jnk, KAT14, Mapk, MEOX2, MGME1, NFkB (complex), NUP42, P glycoprotein, PI3K (complex), PLCL1, RAS, RNA polymerase II, RPS23, SF3B1, TCR, Vegf, ZNF133 | 27 | 13 | Energy Production, Nucleic Acid Metabolism, Small Molecule Biochemistry |
| 2 | Alpha catenin, Ap1, Cdk, CDKN2A, CDKN2B, CDKN2B-AS1, CG, COL1A2, COL3A1, collagen, Collagen Alpha1, Collagen type I (complex), Collagen type III,Collagen(s), Cyclin D, DSTN, ERK1/2, Focal adhesion kinase, HAPLN1, Hsp90, Immunoglobulin, LDL, MAP2K1/2, Mek, MOB4, P38 MAPK, PDGF BB, Ppp2c, RRBP1, Smad, STAT5a/b, TFPI, Tgf beta, VCAN, VEZT | 25 | 12 | Dermatological Diseases and Conditions, Endocrine System Disorders, Organismal Development |
| 3 | 12 lipoxygenase, 3-phosphoglycerate, AGMO, ATP6AP1L, BANF2, beta-estradiol, cholesterol, COQ10B, EGF, ESR2, EZH2, FGD6, GULP1, LILRB5, LINC01016, LINC02085, LRATD1, mir-548, miR-579-3p (and other miRNAs w/seed UCAUUUG), MPV17L, OVOL2, Pat, phosphatidylserine, PXMP4, RB1, SLC10A3, SNX5, TAL1, TCF4, TCHHL1, TGFB1, TOMM7, USP44, Wfdc3, XRCC4 | 25 | 12 | Cellular Development, Cellular Growth and Proliferation, Reproductive System Development and Function |

* Abbreviations: IPA=Ingenuity pathway analysis, HUVEC=human umbilical vein endothelial cell, TAD=topologically associated domain.

**Table S7: IA tissue differential expression studies.***

| **Year** | **1^st^ Author** | **Journal** | **GEO** | **Number of Cases** | **Tissue Source** | **IA vs Ctr.** | **(↑)** | **(↓)** | **R vs UR** | **(↑)** | **(↓)** |
| --- | --- | --- | --- | --- | --- | --- | --- | --- | --- | --- | --- |
| 2019 | Aoki, T. | Scientific Reports | ND | 55 (all UR) † | Clipping | Y | 79 | 329 | N | - | - |
| 2016 | Kleinloog, R. | Stroke | ND | 44 (22 R, 21 UR, 1 uk.) | Clipping | Y | 51 | 178 | Y | 958 | 531 |
| 2009 | Li, L | European Neurology | ND | 3 (all UR) | Clipping | Y**‡** | 164 | 996 | N | - | - |
| 2010 | Pera, J. | Stroke | GSE15629 | 14 (8 R, 6 UR) | Clipping | Y | 33 | 124 | Y | 1 | 31 |
| 2009 | Shi, C | Stroke | ND | 6 (3 R, 3 UR) | Clipping | Y | 172 | 154 | N | - | - |
| 2017 | Wang, W. | Oncotarget | GSE75436 | 27 (12 R, 15 UR) | Clipping | Y | 1511 | 1415 | N | - | - |
| 2011 | Kurki, M.I. | Neurosurgery | ND | 19 (11 R, 8 UR) | Clipping | N | - | - | Y | 686 | 740 |
| 2014 | Nakaoka, H. | Stroke | GSE54083 | 13 (8 R, 5 UR) | Clipping | N | - | - | Y | 430 | 617 |

* †some RNA failed quality, **‡**full gene list not published. Abbreviations: Ctr.=control, IA=intracranial aneurysm, N=no, ND=not disclosed, R=ruptured, uk.=unknown aneurysm status, UR=unruptured, Y=yes.
